# Online Education Plight and Countermeasures for MBBS in Chinese Regional Medical Schools Based on the OBE Concept During COVID-19 Pandemic

**DOI:** 10.3389/fpubh.2021.806809

**Published:** 2022-01-21

**Authors:** Yan Liang, Jingwen Zhang, Ahiafor Maxwell, Chengxia Kan, Ningning Hou, Xiaodong Sun, Zeyun Liu

**Affiliations:** ^1^Department of Biochemistry and Molecular Biology, School of Basic Medicine, Binzhou Medical University, Yantai, China; ^2^Department of Endocrinology and Metabolism, Affiliated Hospital of Weifang Medical University, Weifang, China; ^3^Branch of Shandong Provincial Clinical Research Center for Diabetes and Metabolic Diseases, Weifang, China; ^4^Clinical Research Center, Affiliated Hospital of Weifang Medical University, Weifang, China; ^5^Department of International Student Management, School of International Studies, Binzhou Medical University, Yantai, China

**Keywords:** COVID-19 pandemic, OBE, regional medical schools, MBBS, online education, countermeasures, China

## Introduction

When national borders for studying in China will open, it remains unknown as the coronavirus disease 2019 (COVID-19) pandemic persists. The teaching mode changed from offline to mainly online or a hybrid of the two modes, which generated huge challenges in international education in China. In June 2020, the Ministry of Education issued an announcement emphasizing improvements in education quality and imposing high requirements for international education. Although teachers are unable to teach students face-to-face, they must promote the teaching quality to ensure the reputation and competitiveness of Chinese medical education (1). Outcome-based education (OBE) was proposed by Spady in 1994, which advocates the implementation of teaching activities based on the abilities of students and obtained results by learning, emphasizing student centeredness, and focusing on ability acquisition (2–4). Bachelor of Medicine and Bachelor of Surgery (MBBS) is a highly practical applied major. To obtain medical knowledge, students undergo basic training in the diagnosis, treatment, and prevention of diseases and develop abilities to identify the etiology and pathogenesis of diseases. OBE emphasizes ability training and its core is educational “output” under the guidance of the OBE concept; the exploration of MBBS teaching reform is aimed at integrating ability training into professional education and laying the foundation for the cultivation of high-quality applied medical talents. Although international students in China hail from all over the world and have different ways of thinking, values, and living habits, their attitudes, and understanding of medicine are the same. Regional medical schools are increasingly focusing on improving the quality of international undergraduate education and the OBE concept permeates through the entire process. Schools made many pertinent adjustments during the COVID-19 pandemic, but numerous difficulties remain (5, 6).

## Development of MBBS Education Based on the OBE Concept

With the implementation of “The Belt and Road Initiative” (the Silk Road Economic Belt and the 21st Century Maritime Silk Road unveiled by Chinese President Xi Jinping during his visits to Central and Southeast Asia in September and October 2013), the number of international students in China increased steadily each year. Presently, China has the largest number of international students in Asia. According to relevant statistics, the proportion of academic students reached 54.6% in 2019, which is an increase of 7% from 2016. Students from the countries along the Belt and Road Initiative account for 54.1% of the total, and the number of students who choose to major in MBBS is the largest (7). However, owing to the insufficient attention paid to MBBS, teaching conditions and the quality of faculty in regional medical schools is uneven. Currently, China is experiencing a strategic transition period emphasizing “standardized management, improved quality, and efficiency.” Schools are exploring MBBS reform actively under the OBE concept and taking a series of measures to integrate ability training into professional education and provide support for the cultivation of high-quality applied medical talents (8–12). During the early stages of the COVID-19 pandemic, regional medical schools practiced “classes suspended but learning continues” actively, as the quality of online international education at the time was poor. After the COVID-19 pandemic was effectively controlled in China, classes gradually resumed, moving to “online + offline” teaching. Moreover, students on campus returned to the classroom and those outside China engaged in synchronized online learning.

## Difficulties in MBBS Education Owing to COVID-19 Pandemic

### Lack of Experience of Teachers in English Online Teaching and Confidence in the Process

Currently, more than 70% of students in China are temporarily stranded outside the country and cannot return to school. Thus, implementing normal teaching methods is difficult, especially in courses such as biochemistry, pathology, pathogen biology, immunology, internal medicine, and so on. Before the COVID-19 pandemic, a variety of teaching methods could be adopted flexibly and teachers could adjust their strategies to improve the attention of students. In addition, teaching effects could be tested regularly. By contrast, lacking the assistance of a blackboard for writing and body language, teachers rely only on verbal expressions in online teaching; thus, understanding the learning status of students is difficult. Moreover, concentrating on online classes is difficult for students, resulting in unsatisfactory learning effects. Owing to the short time period for large-scale enrollment, the establishment and operation of online courses are substantial in regional medical schools. English online courses are being developed slowly, leaving only a few resources for students to select. In addition, students outside China are unable to obtain reference books and materials owing to the COVID-19 pandemic.

### Non-integrated Online Teaching Platforms and Various Learning Constraints of Students Outside China

Teachers select online teaching platforms based on their preference including massive open online course (MOOC), Wisdom Tree, Rain Classroom, Superstar Learning Pass, Tencent Classroom, Tencent Meeting, DingTalk, and so on. Although such platforms are convenient for Chinese students, not all are suitable for international students. For example, students outside China are not allowed to register and log into such platforms without a Chinese ID and SIM card and access to certain platforms is restricted in some countries or regions. Therefore, using a unified teaching platform during the COVID-19 pandemic is difficult. Moreover, students outside China face the problem of time differences and are prone to absenteeism and tardiness owing to their inability to adapt to Beijing time. In addition, a reliable network is among the necessary conditions for learning. Some students, especially those in certain African countries, do not have access to reliable networks and, thus, experience network problems such as freezes and disconnections or wired or WiFi networks and use only mobile data. Hence, their willingness to attend classes is reduced significantly owing to high network costs. As a consequence, such students are typically unable to take online classes or log into platforms to watch videos, complete homework, take quizzes, and accomplish other tasks.

### Poor Learning Effect of Experimental Courses Taken by Students Outside China and Inability to Participate in Internships

Experimental courses start as scheduled through major online education modes such as “live broadcast +” “recorded broadcast +,” and “MOOC +.” However, observation and operation are the main class activities. Despite teachers' provision of considerable material and detailed explanations, such contents are not as direct as operational experience. Moreover, audiovisual materials cannot be provided for courses such as regional anatomy, surgery, diagnostics, and medical functional experimentations owing to ethical issues. Thus, students outside China are highly dissatisfied with such courses. Based on the MBBS training program, students begin their internships, combining theoretical, and practical courses, in their fifth academic year. Regional medical schools arrange for students to intern in affiliated hospitals, which is among the long-awaited learning journeys of students. Internships lay the foundation of students for entering hospitals in their final academic year. However, completing internships on the schedule is impossible for students outside China. Some schools uniformly postponed internships for fourth-year students; however, this measure is not a long-term solution.

## Effective Measures That Regional Medical Schools Can Adopt in Response to the Changes

### Improve Teachers' Online Education Abilities Under the OBE Concept

Considering the characteristics of MBBS international students, who are keen on active questioning and extensive research, regional medical schools are attempting to integrate teaching and independent learning organically by changing formats, improving knowledge transfer efficiency, and cultivating students' autonomy in learning. Common teaching methods include team-based learning, case-based learning, problem-based learning, and presentation, assimilation, and discussion. To ensure teaching quality, theoretical course instructors should have solid professional knowledge and rich clinical experience. In principle, such teachers must have more than 1 year of study experience in a native English-speaking country. Internship instructors must be clinicians with an intermediate-level clinician qualification or higher. A hierarchical training model should be adopted by schools to improve teachers' online teaching competencies, including teaching skills, foreign language proficiency, and ability to use multimedia technology. Teachers can observe the teachings of experts from their or other well-known schools, cooperate and exchange with overseas universities, and share educational resources online, which can enable them to obtain guidance from overseas experts. Meanwhile, teaching departments should adhere to the effective traditions of leading newcomers, prepare lessons collectively, and teach demonstratively. Teaching skills competitions should be organized regularly to continuously improve the teaching abilities of young instructors. In addition, teachers should be fully prepared to address potential problems before the start of classes by establishing a sign-in system, providing preview materials, setting up in-class tests, and group discussions, and so on. Moreover, teachers should increase the frequency of video interactions to understand students' attitudes and reflect on teaching effects based on students' opinions and teaching evaluations results.

### Use Online Teaching Platforms Normally and Adopt the “Online + Offline” Blended Teaching Mode

Presently, the blended teaching mode is the most common. A particular platform should be used uniformly and its functions should be adopted as much as possible to reduce the anxiety of students from online platforms. DingTalk stands out among existing platforms, which focus on the co-construction, sharing, application, and integration of high-quality educational resources into teaching, learning, management, and other tasks. Schools should arrange fixed classrooms for online teaching. DingTalk can be downloaded to computers and teachers can hold classes after logging into their accounts. Teachers should prepare PowerPoint courseware to display pictures, animations, videos, and other types of content vividly and intuitively. In class, teachers should pay attention to the status of online students, ask them questions, and get feedback regularly. The preparation of experimental courses is essential in online teaching. For regional anatomy experiments, teachers should provide videos for explaining the entire anatomy process and content and then teach by asking questions. Although online experimental teaching is similar to offline experimental teaching in terms of theoretical knowledge, the interpretation process and visual observation, actual hands-on training, the knowledge acquisition process, and multisensory experiences cannot be obtained and developed solely online. Hence, online experimental teaching should be supplemented once the COVID-19 pandemic is over to compensate for its shortcomings. For internships, roleplaying, which is a new teaching method, should be used to makeup for reduced clinical practice opportunities as well as the comprehensive application of digital resources. The status of students after class should also be determined and questions should be answered in a timely manner. Extracurricular knowledge should be supplemented appropriately, which can serve as an effective measure for face-to-face online teaching. Students outside China should be encouraged to seek hospitals for their internships and the effect can be consolidated through the “online + offline” mode. Furthermore, students must complete their internships and internship tasks in strict accordance with their school's regulations.

### Make Online Teaching and Management Work Together

Cultivating a team of teachers with satisfactory English proficiency to conduct online teaching is necessary and an assessment system must be established by teaching departments. Supervision and inspection should be conducted at the beginning and teaching effectiveness should be assessed at the end of the school year. Supervisory experts, leaders, and instructors should create a specialized group to collect feedback from students and reflect online teaching effects and existing problems in a timely manner. One of the biggest challenges in online education is improving low attendance rates. Teaching departments and instructors should develop an effective attendance management system based on actual conditions, including punishments and linking attendance rates with grades. A formative evaluation combining staged and final assessments should be adopted and the proportion of staged assessment results should be maintained at 60% or higher. Staged assessments include in-class examinations, periodic examinations, laboratory reports, and homework, which are converted into a final score. Formative evaluation can enhance the learning autonomy of students. Additionally, psychological counseling and humanistic education are important for international students. Each student should be taken seriously and effective guidance should be given to those struggling with psychological problems. Teachers can discuss Chinese COVID-19 pandemic prevention measures and the latest policies in class, which can help to alleviate the anxiety of students. Teachers must also bear the burden of conveying the importance of medical skills and ethics to the students to enhance their sense of responsibility and competence.

## Conclusion

Online education accords with the OBE concept and though teaching modes differ, the goals are the same. Ultimately, both methods allow students to acquire knowledge, master skills, and gain expertise. Online teaching is the main method used before restarting the Chinese exchange portal for MBBS. Chinese regional medical schools acquired certain resources and experience since the implementation of online teaching. However, such resources and experience are far from meeting the requirements for efficient and orderly development and increased online education exploration and improvement are necessary. Insufficient online teaching experience, poor classroom conditions, and difficulties in experimental teaching for students outside China are the common online education problems encountered by regional medical schools. Reformation in medical education was stimulated by the COVID-19 pandemic, and online teaching is a powerful accelerator. Schools have become committed to summarizing experiences and lessons from online education by improving management systems to strengthen collaboration and reforming teaching methods to adjust strategies. We should adapt to the changes and seize opportunities during the post-COVID-19 pandemic period, prepare for the establishment of a new online education pattern, and improve MBBS education for international students.

## Author Contributions

ZL and YL conceived the idea, investigated, analyzed the current situation, and wrote the initial draft of the manuscript. CK, NH, JZ, and AM participated in revision, discussion, and presented opinions in this study. XS conceived the idea, and revised the manuscript. All authors contributed to the final manuscript and agreed on the final version of the manuscript.

## Funding

This study was funded by the Shandong Science and Technology Committee (No. ZR2019PH061), the Shandong Medicine and Health Technology Development Project (No. 2018WS553), the Yantai Social Science Planning Research Project (No. YTSK2021-065), and the Quality Improvement of Postgraduate Education in Shandong Province (No. SDYAL19156).

## Conflict of Interest

The authors declare that the research was conducted in the absence of any commercial or financial relationships that could be construed as a potential conflict of interest.

## Publisher's Note

All claims expressed in this article are solely those of the authors and do not necessarily represent those of their affiliated organizations, or those of the publisher, the editors and the reviewers. Any product that may be evaluated in this article, or claim that may be made by its manufacturer, is not guaranteed or endorsed by the publisher.
